# Connective tissue growth factor promotes chemotaxis of preosteoblasts through integrin α5 and Ras during tensile force-induced intramembranous osteogenesis

**DOI:** 10.1038/s41598-021-82246-9

**Published:** 2021-01-27

**Authors:** Wei Jiang, Nobuo Takeshita, Toshihiro Maeda, Chisumi Sogi, Toshihito Oyanagi, Seiji Kimura, Michiko Yoshida, Kiyo Sasaki, Arata Ito, Teruko Takano-Yamamoto

**Affiliations:** 1 Division of Orthodontics and Dentofacial Orthopedics, Graduate School of Dentistry, Tohoku University, Sendai, Miyagi 980-8575 Japan; 2 Department of Pediatrics, Graduate School of Medicine, Tohoku University, Sendai, Miyagi 980-8574 Japan; 3 Department of Biomaterials and Bioengineering, Faculty of Dental Medicine, Hokkaido University, Sapporo, Hokkaido, 060-8586 Japan

**Keywords:** Cell biology, Molecular biology

## Abstract

In vertebrates, new bone formation via intramembranous osteogenesis is a critical biological event for development, remodeling, and fracture healing of bones. Chemotaxis of osteoblast lineage cells is an essential cellular process in new bone formation. Connective tissue growth factor (CTGF) is known to exert chemotactic properties on various cells; however, details of CTGF function in the chemotaxis of osteoblast lineage cells and underlying molecular biological mechanisms have not been clarified. The aim of the present study was to evaluate the chemotactic properties of CTGF and its underlying mechanisms during active bone formation through intramembranous osteogenesis. In our mouse tensile force-induced bone formation model, preosteoblasts were aggregated at the osteogenic front of calvarial bones. CTGF was expressed at the osteogenic front, and functional inhibition of CTGF using a neutralizing antibody suppressed the aggregation of preosteoblasts. In vitro experiments using μ-slide chemotaxis chambers showed that a gradient of CTGF induced chemotaxis of preosteoblastic MC3T3-E1 cells, while a neutralizing integrin α5 antibody and a Ras inhibitor inhibited the CTGF-induced chemotaxis of MC3T3-E1 cells. These findings suggest that the CTGF-integrin α5-Ras axis is an essential molecular mechanism to promote chemotaxis of preosteoblasts during new bone formation through intramembranous osteogenesis.

## Introduction

In vertebrates, new bone formation via intramembranous osteogenesis is a critical biological event for development, remodeling, and fracture healing of bones^1^. At the initial stage of intramembranous osteogenesis, preosteoblasts and their precursors, mesenchymal stem cells, migrate into future bone formation sites from the sites’ surrounding environment^2,3^. The cells differentiate into osteoblasts and initiate secretion of bone extracellular matrices, which are subsequently mineralized, resulting in new bone formation^2^.

Cell migration is divided into two types depending on the directivity of cell movement: random migration and directed migration^4^. Chemotaxis is a type of directed migration; cells migrate toward a gradient of extracellular molecules called chemoattractants^5^. Chemotaxis is an essential cellular process regulating development, homeostasis, wound healing, and cancer metastasis in various tissues, including bones^6^. Chemoattractants bind to specific receptors localized on cell membrane, and the intracellular domain of the receptors activates downstream signaling, which determines cell polarity and stimulates cell motility^7,8^. To date, platelet-derived growth factor (PDGF), insulin-like growth factor 1 (IGF1), bone morphogenetic protein 7 (BMP7), C–C motif chemokine ligand 5 (CCL5), and extracellular calcium are known to work as chemoattractants for preosteoblastic MC3T3-E1 cells, suggesting that they recruit preosteoblasts into sites of new bone formation during intramembranous osteogenesis ^9–12^.

A cysteine-rich matricellular protein connective tissue growth factor (CTGF, also known as CCN2), a member of the CCN (Cry61, CTGF, and Nov) family, is a molecule regulating a number of biological events, such as development, wound healing, and cartinogenesis^13,14^. One cellular process regulated by CTGF during these biological events is cell migration. Previous studies have demonstrated that CTGF induces migration of various cells, including endothelial cells, mesangial cells, and tumor cells^15–17^. Using the Boyden chamber, Ono and colleagues found that bone marrow stem cells migrate toward CTGF, suggesting the chemotactic property of CTGF on osteoblast lineage cells^18^. Integrins, transmembrane heterodimers with α and β subunits, are considered major candidates for receptors of CCN family members^13,14,19^. One integrin that is known to have a significant role in bone formation is integrin α5β1^20,21^. Yang and colleagues analyzed expression of individual integrins in MC3T3-E1 cells by flow cytometry and found a higher intensity of fluorescence labeling integrin α5 than that labeling integrin β1^22^. Integrin α5 has potential to facilitate chemotaxis of cancer cells^23,24^. Importantly, CTGF is a ligand of integrin α5, and the CTGF-induced expression of integrin α5 upregulates adhesion of chondrocytes, suggesting a close relation between CTGF and integrin α5 ^25^. Based on this, we hypothesized that CTGF plays an important role in chemotaxis of preosteoblasts through integrin α5 during intramembranous osteogenesis.

A small GTPase, Ras is ubiquitously expressed in vertebrates and modulates cellular responses to external stimuli^26,27^. It is thought that Ras induces proliferation, differentiation, and gene transcription of osteoblast lineage cells^28,29^. Previous reports showed a close relation between mutations of Ras genes and craniosynostosis, a congenital malformation of cranial bones caused by premature fusion of cranial sutures^30,31^. According to these notions, Ras is considered an important regulator of normal development and growth of bones. As to the role of Ras in chemotaxis, Sasaki and colleagues reported that Ras is highly expressed at the leading edge of migrating *Dictyostelium* cells in response to chemoattractants, resulting in induction of chemotaxis of the cells^32^. Ras is also known to induce chemotaxis of mammalian cells, such as hematopoietic cells and smooth muscle cells^33,34^. Although these findings suggest that Ras plays a key role in promoting chemotaxis, it is still unknown whether Ras exerts the chemotactic properties during development and growth of bones.

The aim of the present study was to evaluate the chemotactic properties of CTGF and its underlying mechanisms during active bone formation through intramembranous osteogenesis. We previously established a mouse tensile force-induced bone formation model in which intramembranous osteogenesis was induced at the osteogenic front of calvarial bones, where bone formation actively occurs, by application of tensile force to calvarial sutures^35^. In the present study, we used the mouse model to analyze the expression and function of CTGF in the recruitment of preosteoblasts at the osteogenic front during active bone formation. We then investigated the chemotactic properties of CTGF on MC3T3-E1 cells using μ-slide chemotaxis chambers that can analyze the effect of chemoattractant gradient on chemotaxis. Moreover, the underlying mechanisms of the CTGF-mediated chemotaxis of MC3T3-E1 cells were explored, focusing on integrin α5 and Ras.

## Results

### Preosteoblasts were aggregated at the osteogenic front of calvarial bones in the mouse tensile force-induced bone formation model

**Figure 1:**
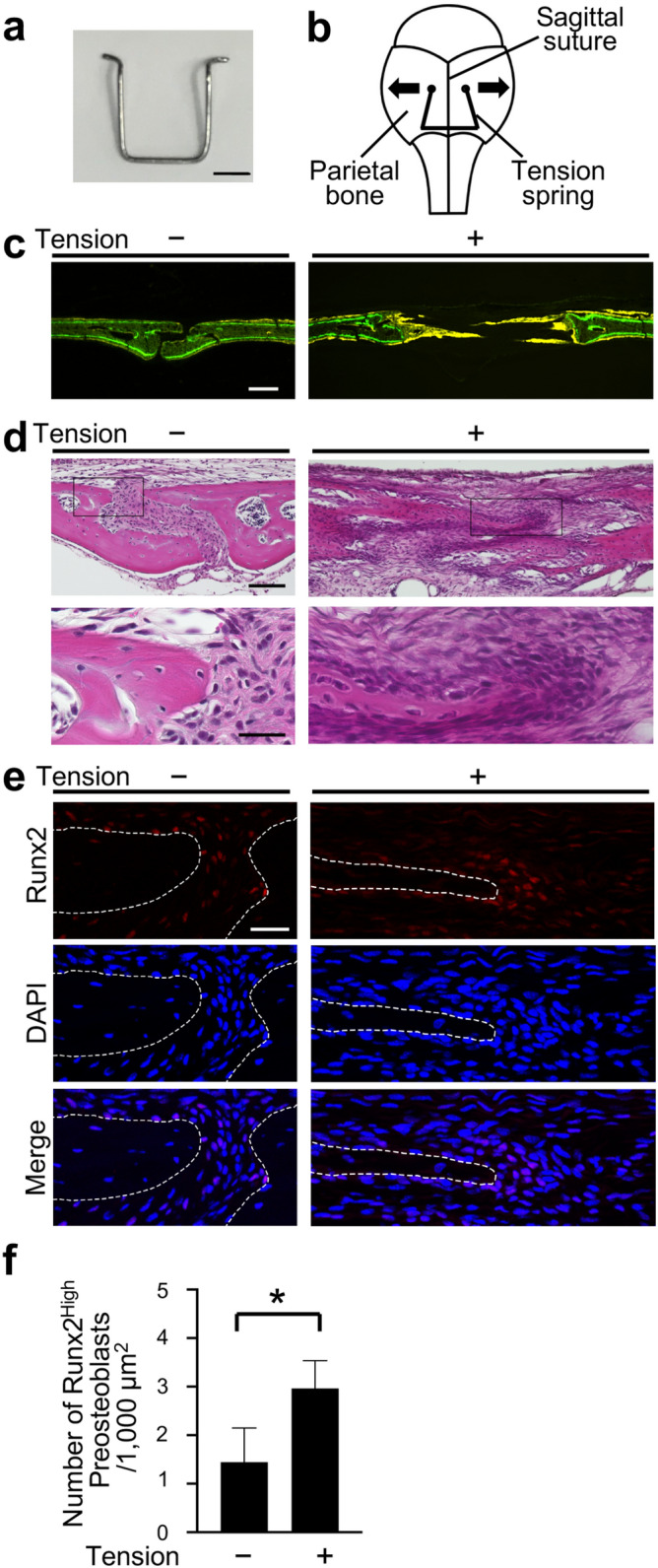
Preosteoblasts were aggregated at the osteogenic front of calvarial bones in the mouse tensile force-induced bone formation model. (**a**) A spring for tensile force loading. Scale bar = 2 mm. (**b**) A schematic diagram of a tension spring placed on the mouse parietal bones. To apply 0.2 N of tensile force to the sagittal suture, a tension spring was set within holes drilled in the right and left parietal bones of 6-week-old mice. Arrows indicate the direction of tension force on the parietal bones. The schematic diagram was created using Affinity Designer (version 1.8.6; https://affinity.serif.com/en-us/designer/). (**c**) Fluorescence micrographs show bone labels with tetracycline (yellow) and calcein (green) in the parietal bones. Scale bar 200 μm. (**d**) The parietal bone and sagittal suture at 7 days after application of tensile force were stained with hematoxylin and eosin. Lower panels show the magnified images of rectangles in upper panels. Scale bar = 100 µm in upper panels, and 30 µm in lower panels. (**e**, **f**) The expression of Runx2 was analyzed by immunohistochemistry (**e**), and the number of Runx2^High^ preosteoblasts at the osteogenic front (**f**) was evaluated (6 animals per group). The dotted line in the image of immunohistochemistry indicates the border of mineralized bone. Scale bar = 30 μm. *p < 0.05, Student’s t-test.

In our mouse tensile force-induced bone formation model (Fig. 1a,b), tensile force continuously expanded the sagittal suture, and the suture width between the right and left parietal bones reached a peak at day 7^35^. Tetracycline-labeled mineralized bone was clearly observed at the edges of the parietal bones at day 7 after tensile force loading, indicating active new bone formation induced by tensile force loading (Fig. 1c). Hematoxylin and eosin (HE) staining showed cell aggregation at the osteogenic front of the newly formed bone in the loaded group at day 7, while it was not observed in the unloaded group (Fig. 1d). No signs of an acute inflammatory response, such as neutrophil infiltration or edema, were shown in the tensile force-loaded sutures (Fig. 1d). Takarada and colleagues reported that preosteoblasts that highly express runt-related transcription factor 2 (Runx2) aggregated at the osteogenic front during calvarial bone development in embryonic mice^36^. We thus analyzed Runx2 expression by immunofluorescence staining and quantified the number of Runx2-highly-expressing (Runx2^High^) preosteoblasts at the osteogenic front in our model. This was 1.45 ± 0.70/1,000 μm^2^ in the unloaded group and a significantly higher 2.96 ± 0.57/1,000 μm^2^ in the loaded group (Fig. 1e,f). These data indicated that preosteoblasts were aggregated at the osteogenic front during tensile force-induced new bone formation of calvarial bones in our mouse model.

### Neutralizing CTGF antibody inhibited the aggregation of preosteoblasts at the osteogenic front in the mouse tensile force-induced bone formation model

**Figure 2:**
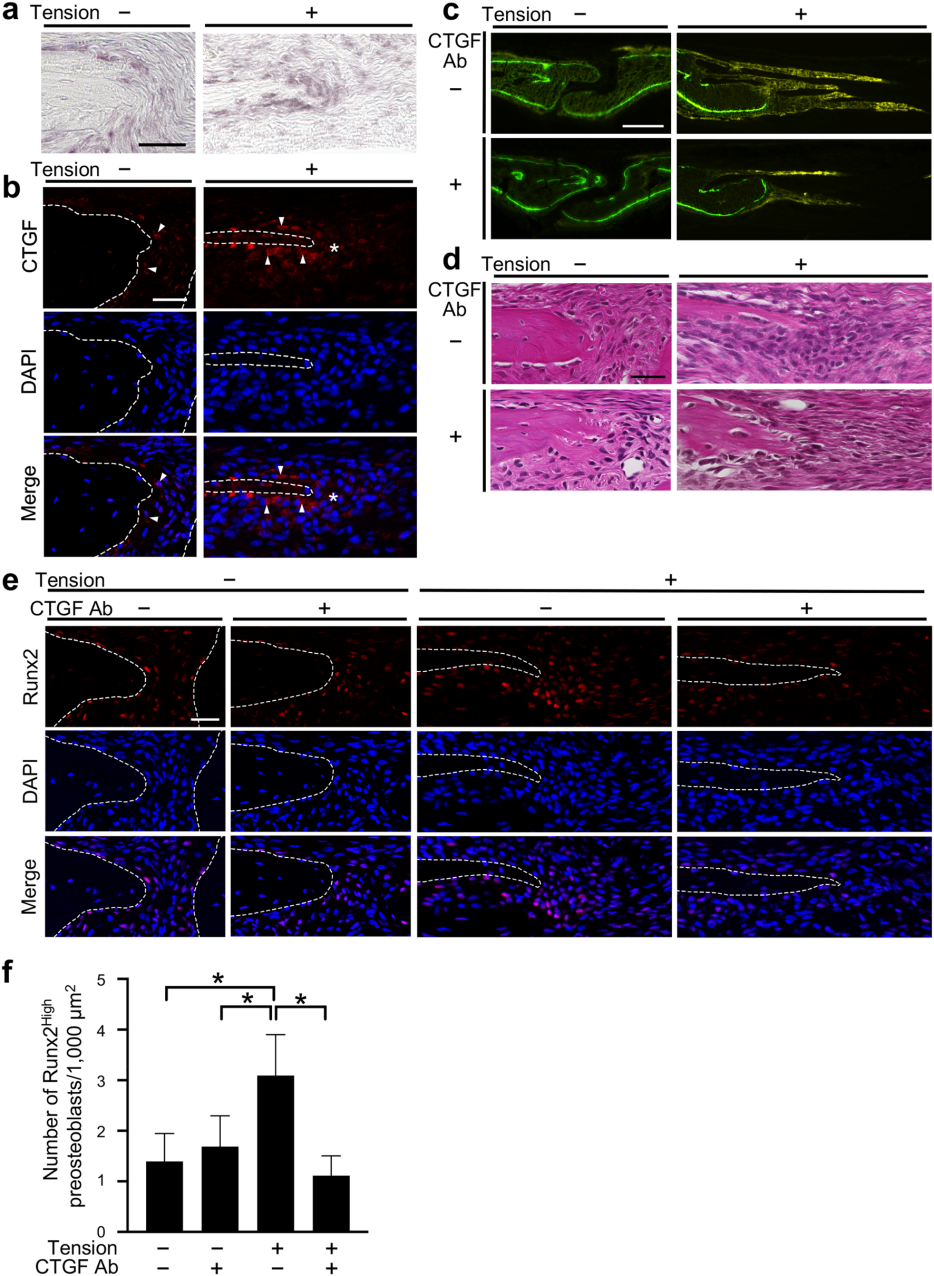
Neutralizing CTGF antibody inhibited the aggregation of preosteoblasts at the osteogenic front in the mouse tensile force-induced bone formation model. (**a**, **b**) Expression of CTGF at the osteogenic front at 7 days after application of tensile force was analyzed by in situ hybridization (**a**) and immunohistochemistry (**b**). The dotted line in the image of immunohistochemistry indicates the border of mineralized bone. Arrowheads indicate CTGF-positive osteoblasts. Asterisks indicate CTGF expression at the osteogenic front. Scale bar = 30 µm. (**c**) Fluorescence micrographs show bone labels with tetracycline (yellow) and calcein (green) at the edges of the parietal bones. A 100 μl volume of 10 μg/ml neutralizing CTGF antibody was injected subcutaneously into the parietal bone area every day during the experimental period. Scale bar 150 μm. (**d**) The parietal bone and sagittal suture at 7 days after application of tensile force without and with the neutralizing CTGF antibody were stained with hematoxylin and eosin. Scale bar 30 μm. (**e**, **f**) The expression of Runx2 was analyzed by immunohistochemistry (**e**), and the number of Runx2^High^ preosteoblasts at the osteogenic front (**f**) was evaluated (6 animals per group). CTGF Ab = neutralizing CTGF antibody. Scale bar = 30 μm. *p < 0.05, ANOVA with Tukey–Kramer post hoc test.

In situ hybridization and immunohistochemistry showed that CTGF was highly expressed in osteoblasts on the surface of the parietal bones in the unloaded group (Fig. 2a,b). In the loaded group, CTGF was expressed at the osteogenic front in addition to in osteoblasts (Fig. 2a,b). We then analyzed function of CTGF in the tensile force-induced bone formation of the parietal bones using a neutralizing CTGF antibody. At day 7, tetracycline-labeled mineralized bone had significantly accumulated at the edges of the parietal bones in the loaded group without the neutralizing CTGF antibody compared to the unloaded groups without and with the antibody (Fig. 2c). The tensile force-induced accumulation of tetracycline-labeled mineralized bone was attenuated by the neutralizing CTGF antibody (Fig. 2c). HE staining showed no apparent difference between without and with the neutralizing CTGF antibody in the unloaded groups (Fig. 2d). Cell aggregation at the osteogenic front was observed in the loaded group without the neutralizing CTGF antibody (Fig. 2d). On the other hand, cell aggregation was not formed in the loaded group with the neutralizing antibody (Fig. 2d). We further examined whether CTGF affected the number of Runx2^High^ preosteoblasts at the osteogenic front during tensile force-induced bone formation. In the unloaded groups, the number of Runx2^High^ preosteoblasts at the osteogenic front without and with the neutralizing CTGF antibody were 1.39 ± 0.55/1,000 μm^2^ and 1.69 ± 0.61/1,000 μm^2^, respectively; there was no significant difference between these groups (Fig. 2e,f). The number in the loaded group without the neutralizing CTGF antibody was 3.09 ± 0.81/1,000 μm^2^; it was significantly higher than in the unloaded groups (Fig. 2e,f). On the other hand, the number in the loaded group with the neutralizing antibody was 1.12 ± 0.39/1,000 μm^2^, significantly lower than in the loaded group without the neutralizing antibody and comparable to the unloaded groups (Fig. 2e,f). To examine whether CTGF directly upregulated Runx2 expression in preosteoblasts, we treated preosteoblastic MC3T3-E1 cells with CTGF. Our data showed that CTGF significantly upregulated Runx2 mRNA expression in MC3T3-E1 cells (Supplementary Fig. S1).

### Proliferation was upregulated at the osteogenic front during tensile force-induced bone formation in a CTGF-independent manner

**Figure 3:**
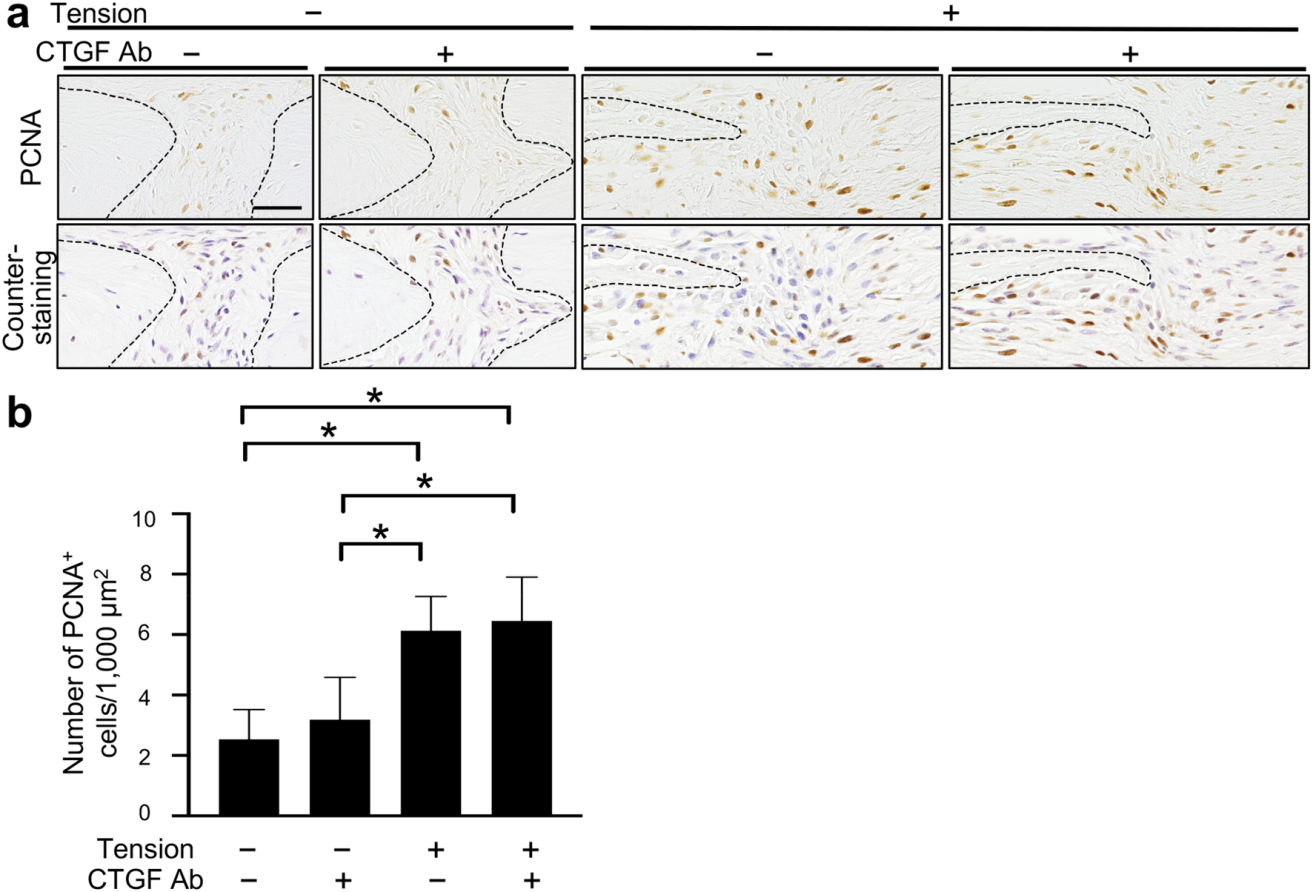
Proliferation was upregulated at the osteogenic front during the tensile force-induced bone formation in a CTGF-independent manner. (**a**) The expression of PCNA at the osteogenic front at 7 days after application of tensile force was analyzed by immunohistochemistry, and counterstaining with hematoxyline was subsequently performed. A 100 μl volume of 10 μg/ml neutralizing CTGF antibody was injected subcutaneously into the parietal bone area every day during the experimental period. The dotted line in the image of immunohistochemistry indicates the border of mineralized bone. (**b**) The number of PCNA^+^ cells at the osteogenic front was evaluated (6 animals per group). CTGF Ab = neutralizing CTGF antibody. Scale bar = 30 μm. *p < 0.05, ANOVA with Tukey–Kramer post hoc test.

To verify if CTGF induced the aggregation of preosteoblasts at the osteogenic front through promotion of proliferation during tensile force-induced bone formation, we next analyzed proliferation by immunohistochemistry of a proliferation marker, proliferating cell nuclear antigen (PCNA). The numbers of PCNA-positive (PCNA^+^) cells at the osteogenic front in the unloaded groups without and with the neutralizing CTGF antibody were 2.54 ± 0.98/1,000 μm^2^ and 3.18 ± 1.40/1,000 μm^2^, respectively; there was no significant difference between these groups (Fig. 3a,b). The numbers were 6.13 ± 1.13/1,000 μm^2^ and 6.45 ± 1.45/1,000 μm^2^ in the loaded groups without and with the neutralizing antibody, respectively; there was no significant difference between these groups, though they were significantly higher than in the unloaded groups (Fig. 3a,b). These data suggested that proliferation of preosteoblasts was upregulated at the osteogenic front during tensile force-induced bone formation in a CTGF-independent manner.

### CTGF gradient induced chemotaxis of preosteoblastic MC3T3-E1 cells

**Figure 4:**
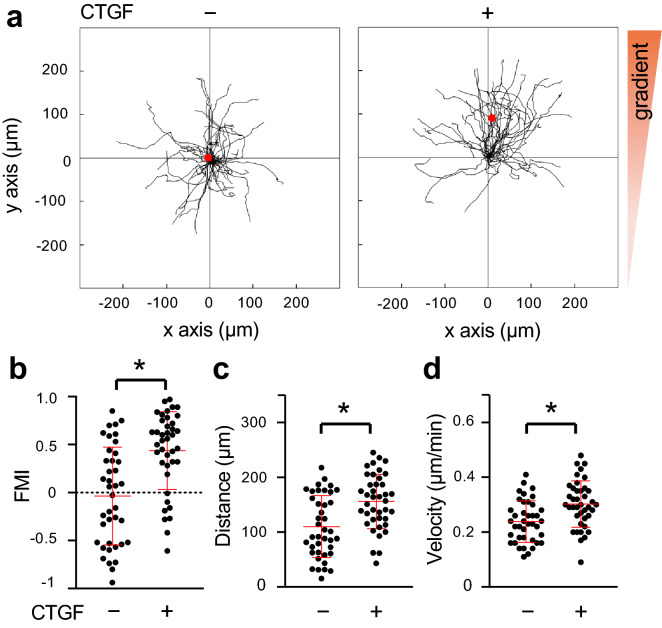
CTGF gradient induced chemotaxis of preosteoblastic MC3T3-E1 cells. (**a**) MC3T3-E1 cells were seeded onto an observation area between two reservoirs in a μ-slide chemotaxis chamber and cultured overnight. Then 150 ng/ml CTGF was added to one reservoir and the cells were allowed to migrate toward CTGF for 12 h. The migration paths of 40 individual cells were analyzed. The red dot represents the center of mass of the endpoints of tracked cells. The parameters FMI (**b**), distance (**c**), and velocity (**d**) were determined (n = 40). *p < 0.05, Mann–Whitney U test.

We examined the chemotactic effect of a gradient of CTGF on preosteoblastic MC3T3-E1 cells using μ-slide chemotaxis chambers. MC3T3-E1 cells moved randomly in all directions in the control group (Fig. 4a). In the CTGF-administered group (CTGF group), on the other hand, the cells migrated toward the gradient of CTGF (Fig. 4a). The forward migration index (FMI) toward the y-axis direction was calculated to quantify the migration of MC3T3-E1 cells toward the gradient of CTGF; FMIs were − 0.03 ± 0.51 and 0.44 ± 0.40 in the control and CTGF groups, respectively (Fig. 4b). Migration distances, defined as direct distance between starting point and end point, were 109.87 ± 55.75 μm and 156.06 ± 49.19 μm in the control and CTGF groups, respectively (Fig. 4c). The velocities (cell migration speeds) were 0.24 ± 0.07 μm/min and 0.30 ± 0.08 μm/min in the control and CTGF groups, respectively (Fig. 4d). In all these parameters, there were significant differences between the control and CTGF groups. These data indicated that the CTGF gradient induced chemotaxis of MC3T3-E1 cells.

### Neutralizing integrin α5 antibody suppressed the CTGF-induced chemotaxis of MC3T3-E1 cells

**Figure 5:**
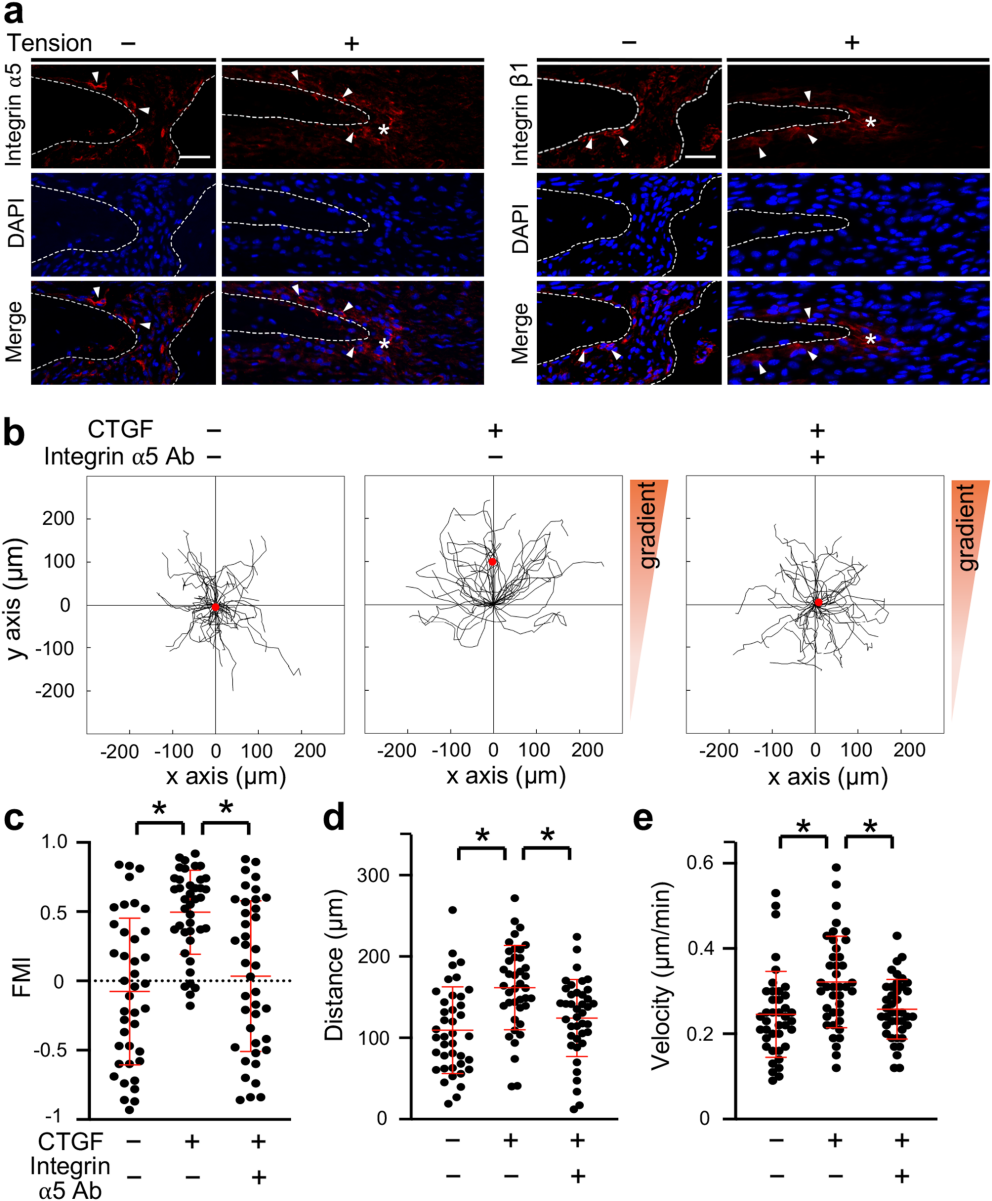
Neutralizing integrin α5 antibody suppressed the CTGF-induced chemotaxis of MC3T3-E1 cells. (**a**) The expression of integrin α5 and integrin β1 at the osteogenic front at 7 days after application of tensile force was analyzed by immunohistochemistry. Arrowheads indicate integrin α5- or integrin β1-positive osteoblasts. Asterisks indicate expression of integrin α5 or integrin β1 at the osteogenic front. The dotted line in the image of immunohistochemistry indicates the border of mineralized bone. Scale bar = 30 μm. (**b**) MC3T3-E1 cells were seeded onto an observation area between two reservoirs in a μ-slide chemotaxis chamber and cultured overnight. The cells in the observation area were pretreated with 5 μg/ml neutralizing integrin α5 antibody for 1 h. Then 150 ng/ml CTGF with or without 15 μg/ml neutralizing integrin α5 antibody was added to one reservoir and the cells allowed to migrate toward CTGF for 12 h. The migration paths of 40 individual cells were analyzed. The red dot represents the center of mass of the endpoints of tracked cells. The parameters FMI (**c**), distance (**d**), and velocity (**e**) were determined (n = 40). Integrin α5 Ab = neutralizing integrin α5 antibody. *p < 0.05, ANOVA with Dunn’s post hoc test.

CTGF activates cell migration and adhesion via binding to integrin α5β1^37–39^. We thus examined integrin α5 and integrin β1 expression at the osteogenic front during tensile force-induced bone formation of the parietal bones. Integrin α5 and integrin β1 were expressed in osteoblasts on the surface of the parietal bones in the unloaded group. In the loaded group, they were expressed in the aggregated cells at the osteogenic front as well as in the osteoblasts on the bone surface (Fig. 5a). We further analyzed the distribution of both CTGF and integrin α5 at the osteogenic front in the loaded group. CTGF was observed in extracellular matrix adjacent to the integrin α5-positive (integrin α5^+^) cells and inside integrin α5^+^ cells (Supplementary Fig. S2). We next analyzed whether integrin α5 regulated the CTGF-induced chemotaxis of MC3T3-E1 cells. MC3T3-E1 cells migrated toward the gradient of CTGF, while a neutralizing integrin α5 antibody inhibited the CTGF-induced directed migration (Fig. 5b). FMI in the CTGF group (0.50 ± 0.30) was significantly higher than in the control group (− 0.08 ± 0.52) (Fig. 5c). FMI in the CTGF and neutralizing integrin α5 antibody-administered group (CTGF-integrin α5 antibody group) (0.02 ± 0.56) was significantly lower than in the CTGF group and comparable to the control group (Fig. 5c). Migration distance in the CTGF group (161.67 ± 51.82 μm) was significantly higher than in the control group (109.47 ± 53.29 μm) (Fig. 5d). Migration distance in the CTGF-integrin α5 antibody group (128.85 ± 55.43 μm) was significantly lower than in the CTGF group and comparable to the control group (Fig. 5d). Velocity in the CTGF group (0.32 ± 0.11 μm/min) was significantly higher than in the control group (0.25 ± 0.10 μm/min), while that in the CTGF-integrin α5 antibody group (0.25 ± 0.08 μm/min) was significantly lower than in the CTGF group (Fig. 5e). We then evaluated the possibility that integrin α5 was related to CTGF-induced Runx2 mRNA expression in preosteoblasts. Our data indicated that the neutralizing integrin α5 antibody did not significantly affect CTGF-induced Runx2 mRNA expression in MC3T3-E1 cells (Supplementary Fig. S3a).

### Ras inhibitor suppressed the CTGF-induced chemotaxis of MC3T3-E1 cells

**Figure 6:**
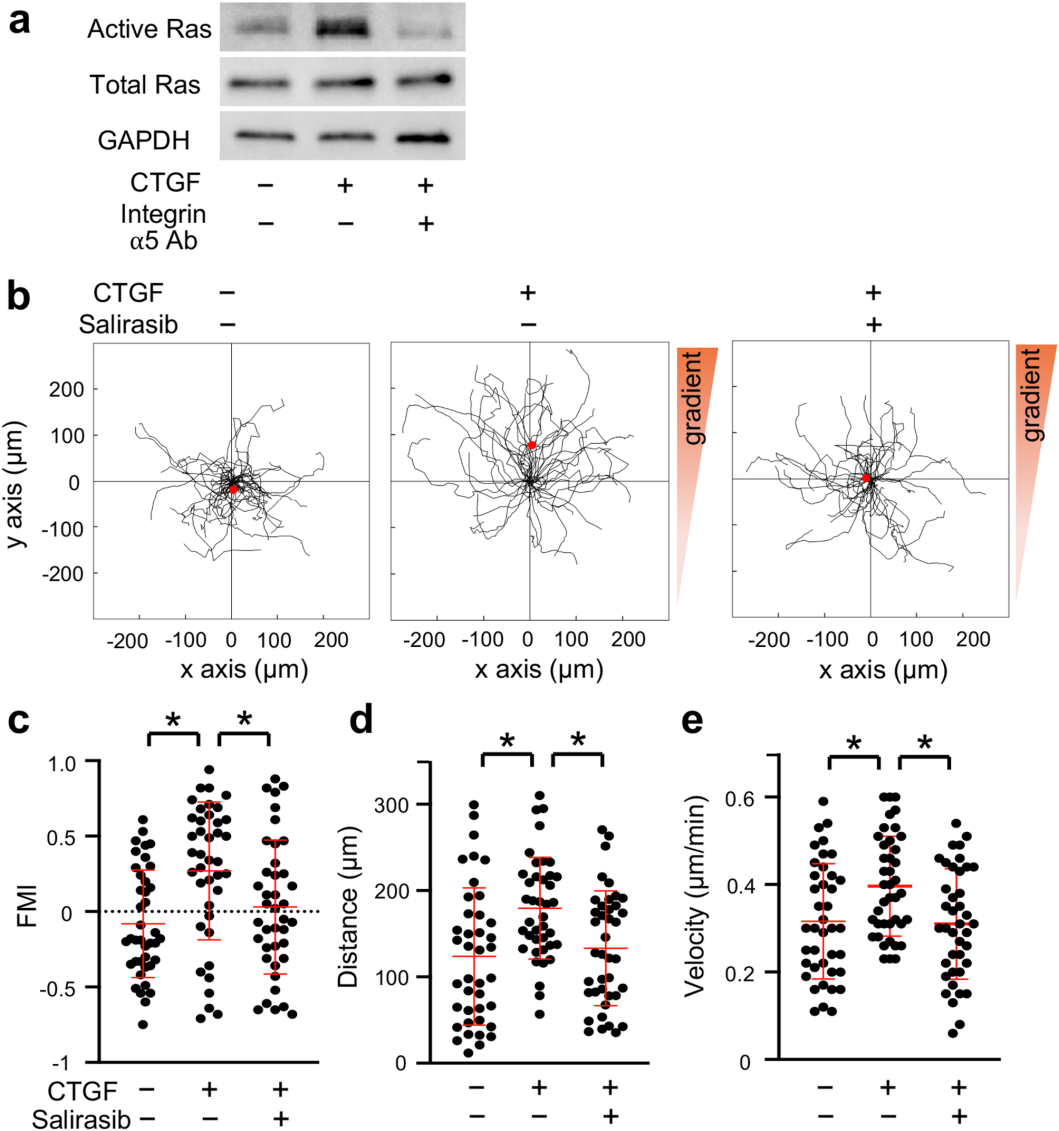
Ras inhibitor suppressed the CTGF-induced chemotaxis of MC3T3-E1 cells. (**a**) MC3T3-E1 cells were seeded onto 10-cm dishes at 45 × 10^4^ cells/dish. Next day, the cells were treated with 50 ng/ml CTGF and 5 μg/ml neutralizing integrin α5 antibody for 12 h. Active Ras was analyzed by immunoblotting of pull-down samples from cell lysates. Full-length blots are presented in Supplementary Fig. S4. (**b**) MC3T3-E1 cells were seeded onto an observation area between two reservoirs in a μ-slide chemotaxis chamber and cultured overnight. The cells in the observation area were pretreated with 10 μM salirasib for 1 h. Then 150 ng/ml CTGF with or without 30 μM salirasib was added to one reservoir and the cells allowed to migrate toward CTGF for 12 h. The migration paths of 40 individual cells were analyzed. The red dot represents the center of mass of the endpoints of tracked cells. The parameters FMI (**c**), migration distance (**d**), and velocity (**e**) were determined (n = 40). Integrin α5 Ab = neutralizing integrin α5 antibody. *p < 0.05, ANOVA with Dunn’s post hoc test.

Ras is a potent inducer of chemotaxis of various types of cells^32–34^. CTGF stimulates migration of epithelial cells via Ras signaling^40^. Ras is a downstream regulator of integrins in osteoblast differentiation^41^. Based on these findings, we focused on Ras as an intracellular regulator of the chemotaxis of MC3T3-E1 cells promoted by CTGF. Ras activity in MC3T3-E1 cells was upregulated by CTGF administration (Fig. 6a). The upregulation of Ras activity was inhibited by the neutralizing integrin α5 antibody (Fig. 6a). Chemotaxis assay showed that MC3T3-E1 cells migrated toward the gradient of CTGF, while the administration of a Ras inhibitor, salirasib, inhibited the CTGF-induced directed movement of MC3T3-E1 cells (Fig. 6b). FMI in the CTGF group (0.27 ± 0.46) was significantly higher than in the control group (− 0.08 ± 0.36), while FMI in the CTGF and salirasib-administered group (CTGF-salirasib group) (0.03 ± 0.44) was significantly lower than that in the CTGF group and comparable to that in the control group (Fig. 6c). Migration distance in the CTGF group (179.52 ± 59.03 μm) was significantly higher than in the control group (123.67 ± 79.48 μm) (Fig. 6d). Migration distance in the CTGF-salirasib group (133.12 ± 66.47 μm) was significantly lower than in the CTGF group; it was comparable to in the control group (Fig. 6d). Velocity in the CTGF group (0.39 ± 0.11 μm/min) was significantly higher than in the control group (0.32 ± 0.13 μm/min); while that in the CTGF-salirasib group (0.30 ± 0.13 μm/min) was significantly lower than in the CTGF group and was comparable to that in the control group (Fig. 6e). We then examined whether CTGF induced Runx2 mRNA expression in preosteoblasts through Ras. Our data indicated that salirasib did not clearly affect CTGF-induced Runx2 mRNA expression in MC3T3-E1 cells (Supplementary Fig. S3b).

## Discussion

The recruitment of preosteoblasts to future bone formation sites is an important cellular process for normal bone formation during bone development, regeneration, and fracture healing through intramembranous osteogenesis^2,3^. Therefore, identification of its regulatory factors and the elucidation of their molecular biological mechanisms are critical issues in bone biology. In the present study, we hypothesized that CTGF is a novel chemoattractant to facilitate the chemotaxis of preosteoblasts at the sites of new bone formation during intramembranous osteogenesis. To evaluate this hypothesis, we analyzed the expression and function of CTGF at the osteogenic front in our mouse tension force-induced bone formation model. We also examined the chemotactic effect of CTGF on MC3T3-E1 cells and its underlying mechanisms using μ-slide chemotaxis chambers.

We previously analyzed time-dependent bone formation of the parietal bones for 28 days using the same mouse model as that in the present study. Micro computed tomography (CT) analysis revealed that the suture width between the edges of the right and left parietal bones reached a peak at day 7 after tensile force loading^35^. Histological analysis showed a higher degree of bone formation at the sutural bony edges at day 7 compared to other time points without obvious signs of an acute inflammatory response^35^. Based on these previous data, we analyzed the expression of Runx2^High^ preosteoblasts at day 7 in our mouse tensile force-induced bone formation model in the present study. At this time point, aggregation of Runx2^High^ preosteoblasts was observed at the osteogenic front of calvarial bones. During formation of calvarial bones in embryonic mice, preosteoblasts that highly express Runx2 aggregated at the osteogenic front^36^. These findings indicated that the aggregation of preosteoblasts at the osteogenic front during calvarial bone growth under the physiological conditions was replicated in our model. We used the mouse model to investigate the role of CTGF in the aggregation of preosteoblasts during active bone formation.

We found that CTGF mRNA and protein were expressed at the osteogenic front where preosteoblasts aggregated during tensile force-induced bone formation of calvarial bones, indicating that the aggregated preosteoblasts produced CTGF during active bone formation. Our immunohistochemical data showed CTGF distribution inside integrin α5^+^ cells at the osteogenic front. Integrin signaling induces CTGF expression in myoblastic C2C12 cells^42^. CTGF upregulates integrin α5 expression in chondrocytes^25^. These findings suggest that there is a positive feedback loop between CTGF and integrin α5 production in the aggregated preosteoblasts at the osteogenic front during intramembranous osteogenesis. Transforming growth factor-β (TGF-β) regulates bone formation promoting migration and proliferation of osteoblast precursors^43,44^. It is well known that TGF-β potently stimulates CTGF transcription via binding of its downstream molecules, such as Smad, to specific regulatory binding elements in the CTGF promoter^45^. Moreover, CTGF and TGF-β1 are co-expressed in mesenchymal condensation that is observed in early cartilage formation during endochondral ossification^46^. Therefore, it seems likely that TGF-β is involved in the regulatory mechanism of CTGF in the aggregated preosteoblasts during the tensile force-induce bone formation of calvarial bones. In the present study, suppression of CTGF function by the neutralizing antibody resulted in inhibition of tensile force-induced bone formation. Importantly, the aggregation of Runx2^High^ preosteoblasts at the osteogenic front was not observed in the tensile force-loaded group with administration of the neutralizing CTGF antibody. These results suggest that CTGF promotes the aggregation of preosteoblasts and subsequent new bone formation at the osteogenic front. At the initial stage of bone formation of calvarial bones, preosteoblasts aggregate at the osteogenic front through recruitment and proliferation of the cells^2,3^. Because CTGF is a potent inducer of proliferation of osteoblast lineage cells^47^, we examined whether CTGF facilitated the aggregation of preosteoblasts at the osteogenic front through upregulation of proliferation. Our data showed that the neutralizing CTGF antibody did not obviously affect proliferation of cells at the osteogenic front under tensile force loading, suggesting that the CTGF-induced aggregation of preosteoblasts at the osteogenic front was not promoted by the proliferation of preosteoblasts. Therefore, we next examined the possibility that CTGF induced the aggregation of preosteoblasts through recruitment of cells into the osteogenic front during new bone formation.

The Boyden chamber has been conventionally used for in vitro analysis of chemotactic ability of molecules. However, the Boyden chamber has a limitation: it cannot generate a gradient of chemoattractants^48^. Because chemotaxis is triggered by cells sensing a gradient of chemoattractants in surrounding environments^5^, we used μ-slide chemotaxis chambers, which overcome the limitation of the Boyden chamber, to examine the effect of the CTGF gradient on chemotaxis of MC3T3-E1 cells. Our data showed an increase in FMI in the CTGF group, indicating that MC3T3-E1 cells migrated toward the CTGF gradient. This result suggests that CTGF expressed at the osteogenic front works as a chemoattractant for preosteoblasts and that the CTGF-induced chemotaxis of preosteoblasts causes the aggregation of the cells that is observed in our mouse tensile force-induced bone formation model. Moreover, our data showed that migration distance and velocity were upregulated in the CTGF-induced chemotaxis of MC3T3-E1 cells. These data suggest that CTGF regulates the efficiency, as well as the directivity, of preosteoblast migration. The increased efficiency of migration of preosteoblasts by CTGF may contribute to the cells’ rapid aggregation at the sites of bone formation in response to the gradient of CTGF during active bone formation.

Chemotaxis is activated through binding of extracellular chemoattractants to transmembrane receptors and subsequent upregulation of intracellular signaling. Integrins are considered to be functional receptors of CTGF^13,14^. In the present study, we found that integrin α5 was expressed at the osteogenic front during active bone formation in our mouse model. Moreover, our immunohistochemical data showed that integrin α5^+^ cells were adjacent to CTGF in extracellular matrix at the osteogenic front. Based on these findings, we examined whether CTGF exerts chemotactic activity through integrin α5 in MC3T3-E1 cells. Our data indicated that the neutralizing integrin α5 antibody suppressed the CTGF-induced chemotaxis of MC3T3-E1 cells. This result suggests that CTGF induces chemotaxis of preosteoblasts toward bone formation sites through integrin α5. CTGF protein consists of 4 modules that bind to other molecules: an IGF-binding protein-like module, a von Willebrand type C repeat module, a thrombospondin type 1 repeat module, and a C-terminal (CT) module^49^. The neutralizing CTGF antibody used in the present study binds to the CT module. It is known that the CT module of CTGF binds to integrin α5β1^37–39^. This suggests that CTGF binds to integrin α5 via the CT module and triggers the chemotaxis of preosteoblasts.

Our data showed that CTGF upregulated Ras activity in MC3T3-E1 cells, while the neutralizing integrin α5 antibody suppressed the CTGF-induced Ras activity. Moreover, the Ras inhibitor salirasib inhibited the CTGF-induced chemotaxis of MC3T3-E1 cells. These data indicated that Ras is an intracellular downstream regulator of the CTGF-integrin α5 signaling which induces chemotaxis of MC3T3-E1 cells. This is the first biological evidence revealing that Ras exerts chemotactic properties in osteoblast lineage cells. To date, details of the integrin α5-Ras pathway in preosteoblast chemotaxis is not understood; nevertheless, focal adhesion kinase (FAK) may be involved in the pathway. FAK, a cytoplasmic tyrosine kinase, is a key regulator of migration of various cells including bone marrow stem cells^50,51^. FAK is known to be a downstream regulator of integrins, including integrin α5^52,53^. Integrin-FAK stimulates Ras signaling and promote biological processes such as cell survival, differentiation, proliferation, and tumorigenesis^41,54,55^.Therefore, we speculate that the integrin α5-FAK-Ras pathway is a regulatory mechanism of preosteoblast chemotaxis. Moreover, Ras positively and negatively regulates integrin activity depending on biological contexts^56^. This indicates a possibility that there is a feedback regulation between integrin α5 and Ras in preosteoblast chemotaxis. As well as Ras, other small GTPases, Rac1 and Cdc42, are expressed at the leading edge of migrating cells and regulate chemotaxis^8^. It is known that CTGF induces activity of Rac1 and Cdc42 in keratinocytes and umbilical vein endothelial cells^57,58^. According to these findings, in addition to Ras, Rac1 and Cdc42 may be involved in the CTGF-induced chemotaxis. We need further investigation to elucidate the molecular mechanism of CTGF-induced preosteoblast chemotaxis.

Our data showed that CTGF upregulated Runx2 expression in MC3T3-E1 cells. Other groups have also shown CTGF-induced Runx2 expression in MC3T3-E1 cells and mouse primary osteoblasts^59,60^. These findings suggest that CTGF enhances bone formation, inducing osteoblast differentiation of preosteoblasts through induction of Runx2 expression, as well as chemotaxis of preosteoblasts, during intramembranous osteogenesis. We further analyzed the effect of integrin α5 and Ras on the CTGF-induced Runx2 expression in MC3T3-E1 cells. Our data showed that the neutralizing integrin α5 antibody and salirasib did not significantly affect the induction of Runx2 expression by CTGF. It has been reported that integrin α5 and Ras have the potential to induce osteoblast differentiation^41,61^; however, our data suggested that they are not responsible for CTGF-induced Runx2 expression in preosteoblasts.

In conclusion, the present study revealed that preosteoblasts aggregate at the osteogenic front during the tensile force-induced bone formation of calvarial bones. CTGF is expressed at the osteogenic front and functional inhibition of CTGF using neutralizing antibody suppresses the aggregation of preosteoblasts. In vitro experiments using μ-slide chemotaxis chambers showed that CTGF induces chemotaxis of MC3T3-E1 cells, while the neutralizing integrin α5 antibody and Ras inhibitor inhibit the CTGF-induced chemotaxis of MC3T3-E1 cell. These findings suggest, for the first time, that the CTGF-integrin α5-Ras axis is an essential molecular mechanism to promote chemotaxis of preosteoblasts during new bone formation through intramembranous osteogenesis. It has been reported that CTGF plays a critical role in intramembranous osteogenesis through induction of proliferation and differentiation of osteoblast lineage cells, and secretion and mineralization of bone matrix^62–64^. The present study sheds light on the role of CTGF as a chemoattractant for osteoblast lineage cells in intramembranous osteogenesis.

## Methods

### Ethics statement

Institute of Cancer Research (ICR) mice were purchased from CLEA Japan (Tokyo, Japan). Animal experiments were performed in accordance with the Regulations for Animal Experiments and Related Activities at Tohoku University. All animal protocols were approved by the Institutional Animal Care and Use Committee of the Tohoku University Environmental and Safety Committee. We complied with the ARRIVE guidelines (Animal Research: Reporting of In Vivo Experiments).

### Application of tensile force to the mouse sagittal suture

Application of tensile force to the mouse sagittal suture was performed according to our previous report^35^. Six-week-old male ICR mice were anesthetized by intraperitoneal injection of medetomidine (0.3 mg/kg), midazolam (4 mg/kg), and butorphanol (5 mg/kg). A skin incision was made to expose the parietal bones. Two holes were made equidistant from a sagittal suture at the anteroposterior middle of the parietal bones using a round bur attached to a dental drill. The distance between the two holes was 3 mm. Tension springs delivering tensile force to the sagittal suture were formed with 0.3 mm diameter orthodontic nickel-titanium wire (Fig. 1a). The springs were calibrated to load an initial 0.2 N of tensile force onto the sagittal sutures prior to each experiment. The spring was set within the holes in the parietal bones (Fig. 1b) and fully covered by the skin; the incision was closed by suturing. In the unloaded group, identical surgical procedures were performed without spring installation. For functional inhibition of CTGF, a 100 μl volume of 10 μg/ml neutralizing CTGF antibody (PeproTech, Rocky Hill, NJ, USA) in saline was subcutaneously injected into the sagittal suture area 6 h before the tension spring was set. The antibody was thereafter injected every day during the experimental period. In the control group, a 100 μl volume of 10 μg/ml rabbit IgG (Sigma-Aldrich, St. Louis, MO, USA) in saline was injected. During all of the experiments, mice were carefully monitored and no adverse events were observed.

### Analysis of new bone formation

Calcein (20 mg/kg) (Dojindo, Kumamoto, Japan) and tetracycline (20 mg/kg) (Wako, Osaka, Japan) were administered into mice by intraperitoneal injection at 1 day before application of tensile force and sacrifice, respectively. The calvaria dissected at day 7 after tensile force loading were embedded in 4% carboxymethyl cellulose sodium salt (Section-Lab, Hiroshima, Japan) and frozen. Undecalcified sections at a thickness of 8 μm were obtained according to Kawamoto’s film method^65^. Images of the sections were acquired using a fluorescence microscope (BZ-9000; Keyence, Osaka, Japan).

### HE staining

Mice were perfusion fixed with 4% paraformaldehyde (PFA) (Sigma-Aldrich) in phosphate buffered saline (PBS) under inhalation anesthesia with isoflurane after application of the tensile force for 7 days. The sagittal suture and parietal bone were dissected and immersed in 4% PFA in PBS at 4 °C overnight. The specimens were decalcified with 20% ethylenediaminetetraacetic acid (pH 7.4) at 4 °C for 1 week. The specimens were then embedded in paraffin, sectioned at 5 μm, and stained with hematoxylin (Sigma-Aldrich) and eosin (Wako).

### In situ hybridization

In situ hybridization was performed according to our previous report with some modifications^66^. Digoxigenin-labelled RNA probes were prepared with the MAXIscript Kit (Thermo Fisher, Waltham, MA, USA), in accordance with the manufacturer’s instructions. The DNA fragment of CTGF (NM_010217, located between 2252 and 2372) was subcloned into the pGEM-T Easy vector (Promega, Madison, WI, USA) and used to generate sense and antisense probes. Sections were deparaffinized and incubated with 3 μg/ml proteinase K (Roche, Mannheim, Germany) for 15 min at 37 °C. After fixation and prehybridization, sections were hybridized overnight at 42 °C with digoxigenin-labelled RNA probes. RNase A treatment (20 μg/ml; Roche) was carried out at 37 °C for 30 min. The sections were incubated with 1.5% blocking reagent (Roche) for 60 min at room temperature and then with anti-digoxigenin antibody conjugated with alkaline phosphatase (Roche) for 40 min at room temperature. Nitro blue tetrazolium and 5-bromo-4-chloro-3-indolyl phosphate (Roche) were used for signal detection.

### Immunohistochemistry

Immunohistochemistry was performed according to our previous study^67^. Deparaffinized sections were immersed in 0.01 M citrate solution and microwaved for 2 min, then incubated at room temperature for 30 min. The sections were treated with 10% donkey serum (Sigma-Aldrich) in PBS at room temperature for 1 h and incubated with rabbit anti-Runx2 (1:1600; Cell Signaling Technology, Danvers, MA, USA), rabbit anti-CTGF (1:800; Abcam, Cambridge, England), goat anti-CTGF (1:100; LifeSpan BioSciences, Seattle, WA, USA), rabbit anti-integrin α5 (1:100; Abcam), or rabbit anti-integrin β1 (1:200; Abcam) antibodies at 4 °C overnight. In the negative controls, the primary antibodies were omitted. After washing with PBS, the sections were incubated with donkey anti-goat-IgG Alexa Fluor 488 and anti-rabbit-IgG Alexa Fluor 568 (1:500; Invitrogen, Carlsbad, CA, USA) at RT for 1 h. 4′,6-diamidino-2-phenylindole (DAPI; SeraCare, Milford, MA, USA) was used for nuclei detection. Fluorescent signals were visualized using a confocal laser scanning microscope system (C2si; Nikon, Tokyo, Japan).

For the detection of PCNA, deparaffinized sections were treated with 3% H_2_O_2_ in methanol and incubated overnight at 4 °C with a rabbit anti-PCNA antibody (1:2000; Abcam) in Can Get Signal immunostain Solution B (Toyobo, Osaka, Japan). After incubation with a peroxidase-conjugated secondary antibody (Histofine Simple Stain Mouse MAX PO; Nichirei, Tokyo, Japan) at room temperature for 30 min, the signals were visualized with 3,3-diaminobenzi-dine tetrahydrochloride (DAB; Nichirei).

### Histomorphometric analysis

To quantify the number of Runx2^High^ preosteoblasts at the osteogenic front, the fluorescent images were analyzed using ImageJ software (NIH, Bethesda, MD, USA; https://imagej.nih.gov/ij/); thresholds of 0 and 235 were used in red channel images. Expression level of Runx2 in osteoblast lineage cells is increased as osteoblast differentiation advances^36^. That is, mesenchymal stem cells in intrasutural mesenchyme express Runx2 at low levels, while preosteoblasts at the osteogenic front and osteoblasts at bone surface express Runx2 at high levels^36^. Based on this knowledge, we determined the thresholds at which signals were detected in preosteoblasts in the osteogenic front but not in mesenchymal stem cells in a suture region, by superimposition of the binary images with the corresponding fluorescent images. The images obtained from PCNA immunohistochemistry were also analyzed using ImageJ; thresholds of 0 and 180 were used to quantify the PCNA^+^ cells.

The two square fields, which were used as the regions of interest (ROI, 50 × 50 μm), were placed on the right and left osteogenic front regions, so that the center of the square field was right on the tip of the parietal bone adjacent to the sagittal suture. The area of the osteogenic front in a suture region was obtained by subtraction of the bone area inside the ROI from the area of the ROI (2,500 μm^2^). The number of Runx2^High^ preosteoblasts and PCNA^+^ cells in the area of the osteogenic front was represented as a cell number per 1,000 μm^2^. The average number of cells in the two ROIs was used as the value of each sample. We used six animals per group and confirmed the sample size was sufficient for this study (the powers of sample were more than 0.8) using G*Power 3.1 software introduced by Faul et al^68^.

### Cell culture

For Ras activation assay, MC3T3-E1 cells were seeded onto 10-cm dishes at 45 × 10^4^ cells/dish and cultured in Minimum Essential Medium Eagle Alpha Modification (α-MEM; Wako) supplemented with 10% fetal bovine serum (FBS) (Hyclone, Logan, UT, USA), 100 units/ml penicillin, and 100 μg/ml streptomycin (Thermo Fisher). After 24 h of culture, the medium was replaced by a medium containing 5 μg/ml neutralizing integrin α5 antibody (SouthernBiotech, Birmingham, AL, USA) for pretreatment of the cells with the antibody for 1 h. Then, recombinant CTGF (ProSpec, Rehovot, Israel) was added to the medium containing the antibody to a final concentration of 50 ng/ml and the cells were cultured for another 12 h. The cells were lysed using CelLytic M Lysis Reagent (Sigma-Aldrich) containing Protease Inhibitor Cocktail (Sigma-Aldrich). For analysis of Runx2 expression by real-time polymerase chain reaction (PCR), MC3T3-E1 cells were seeded onto 24-well plates at 2 × 10^4^ cells/well. When the cells reached confluence, the medium was replaced by α-MEM supplemented with 0.5% FBS, 50 ng/ml CTGF, and either 5 μg/ml neutralizing integrin α5 antibody or 10 μM Ras inhibitor salirasib (Sigma-Aldrich). After 3 days of culture, cells were harvested to isolate total mRNA using RNeasy Kit (QIAGEN, Venlo, Netherlands) according to the manufacturer’s protocol. Each experiment was performed independently three times.

### Ras activation assay

Ras activities were analyzed using the Ras Activation Assay Kit (Millipore, Billerica, MA, USA), according to the manufacturer’s instructions with some modifications. Active Ras was pulled down with Raf-1 Ras binding domain (RBD)-conjugated agarose beads (Millipore) at 4 °C for 1 h. The protein samples were boiled in Laemmli (Bio Rad, Hercules, CA, USA) for 5 min. Sodium dodecyl sulfate (SDS) polyacrylamide gel electrophoresis was performed, then the proteins were transferred to polyvinylidene fluoride membranes (Bio Rad). The membranes were treated with Block Ace (KAC, Kyoto, Japan) for 1.5 h and incubated overnight at 4 °C with mouse anti-Ras (1:500; Millipore) or mouse anti-glyceraldehyde-3-phosphate dehydrogenase (GAPDH; 1:2500; Proteintech, Rosemont, IL, USA) antibodies. The membranes were then incubated with horseradish peroxidase-conjugated secondary antibodies, and developed with EzWestLumi One (Atto, Tokyo, Japan). Chemiluminescent signals were acquired using the Fusion FX Imaging System (Vilber Lourmat, Marne La Vallée, France).

### Real-time PCR

Real-time PCR was performed according to our previous study^67^. cDNA was synthesized from 0.6 μg of total mRNA using a PrimeScript RT Reagent Kit (Takara Bio, Shiga, Japan). Real-time PCR was performed using a Thermal Cycler Dice Real Time System (Takara Bio). The reaction volume was 25 μl, which contained 2 μl of cDNA, 12.5 μl of TB Green Premix Ex Taq II (Takara Bio), and 0.4 μM of sense and antisense primers. The primer sequences are listed in Supplementary Table S1. The reactions consisted of 40 cycles of 5 s at 95 °C and 30 s at 60 °C. Relative expression of mRNA was normalized to that of GAPDH and analyzed by the ΔΔCT method.

### Chemotaxis assay

Chemotaxis of MC3T3-E1 cells was analyzed using μ-slide chemotaxis chambers (Millipore) coated with 0.15 mg/ml collagen type I (Nitta Gelatin, Osaka, Japan). MC3T3-E1 cells were trypsinized and resuspended with α-MEM supplemented with 10% FBS, 100 units/ml penicillin, and 100 μg/ml streptomycin at a density of 3 × 10^6^ cells/ml; a narrow observation area between two reservoirs in the chemotaxis chamber was filled with the cell suspension (6 μl). After 6 h of culture, the cells were treated with 12 μg/ml Mitomycin C (Kyowa Hakko, Tokyo, Japan) for 2 h to inhibit cell proliferation, and washed and incubated with the medium overnight. To examine the effect of the neutralizing integrin α5 antibody or salirasib on the CTGF-modulated chemotaxis of MC3T3-E1 cells, the cells were pretreated with these reagents for 1 h. Then, one of the reservoirs was filled with α-MEM containing 10% FBS and the other was filled with α-MEM containing 10% FBS and 150 ng/ml CTGF with 15 μg/ml neutralizing integrin α5 antibody or 30 μM salirasib. The chamber was mounted on a Celldiscoverer 7 (Zeiss, Jena, Germany) and serial images of cells in the observation area of the chamber were captured every 15 min for 12 h. After the live-cell imaging, 40 cells in the image captured at the start of observation were randomly selected and used for tracking cell movement over 12 h and calculation of the parameters (center of mass, FMI, distance, and velocity) using Manual Tracking and Chemotaxis Tool plugins in ImageJ. Each experiment was performed independently three times.

### Statistical analysis

Prism 8 (GraphPad Software, San Diego, CA, USA) was used for statistical analysis. Data of the quantification of immunohistochemistry were analyzed by Student’s t-test or an analysis of variance (ANOVA) with the Tukey–Kramer post hoc test. Chemotaxis data were analyzed by the Mann–Whitney U test or ANOVA with the Dunn’s post hoc test. P values less than 0.05 were considered statistically significant. All data are presented as mean ± standard deviation (SD).

## Supplementary Information

Supplementary Information.

## Data Availability

The data that support the findings of this study are available from the corresponding author upon reasonable request.
