# Supplementary material for: Connective tissue growth factor promotes chemotaxis of preosteoblasts through integrin α5 and Ras during tensile force-induced intramembranous osteogenesis
Source: Sci Rep. 2021 Jan 27;11:2368. doi: 10.1038/s41598-021-82246-9 (PMC7841149; doi:10.1038/s41598-021-82246-9)
Supplement: Supplementary file 1 — Supplementary Information. [file 41598_2021_82246_MOESM1_ESM.docx]

**Connective tissue growth factor promotes chemotaxis of preosteoblasts through integrin α5 and Ras during tensile force-induced intramembranous osteogenesis**

Wei Jiang^1^*, DDS; Nobuo Takeshita^1^*, DDS, PhD; Toshihiro Maeda^1^, DDS, PhD; Chisumi Sogi^2^, MD, PhD; Toshihito Oyanagi^1^, DDS, PhD; Seiji Kimura^1^, DDS, PhD; Michiko Yoshida^1^, DDS, PhD; Kiyo Sasaki^1^, DDS, PhD; Arata Ito^1^, DDS, PhD; Teruko Takano-Yamamoto^1,3^**, DDS, PhD

1 Division of Orthodontics and Dentofacial Orthopedics, Graduate School of Dentistry, Tohoku University, Sendai, Miyagi, 980-8575, Japan.

2 Department of Pediatrics, Graduate School of Medicine, Tohoku University, Sendai, Miyagi, 980-8574, Japan.

3 Department of Biomaterials and Bioengineering, Faculty of Dental Medicine, Hokkaido University, Sapporo, Hokkaido, 060-8586, Japan.

*These authors contributed equally to this work.

**Corresponding author: Teruko Takano-Yamamoto, DDS, PhD, Division of Orthodontics and Dentofacial Orthopedics, Tohoku University Graduate School of Dentistry, 4-1, Seiryomachi, Aoba-ku, Sendai, 980-8575, Japan

Tel: +81 22 7178374, Fax: +81 22 7178378, E-mail: t-yamamo@m.tohoku.ac.jp

**Supplementary Information**


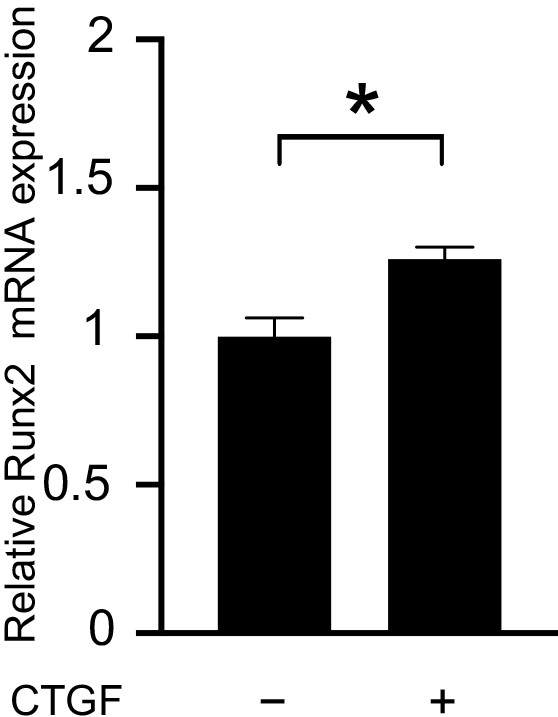


**Supplementary Figure S1. CTGF induced Runx2 mRNA expression in MC3T3-E1 cells.** MC3T3-E1 cells were seeded onto 24-well plates at 2 × 10^4^ cells/well. The cells were treated with 50 ng/ml CTGF for 3 days after they reached confluence. The mRNA expression of Runx2 was analyzed by real-time PCR (n=3). *p < 0.05, Student’s t-test.


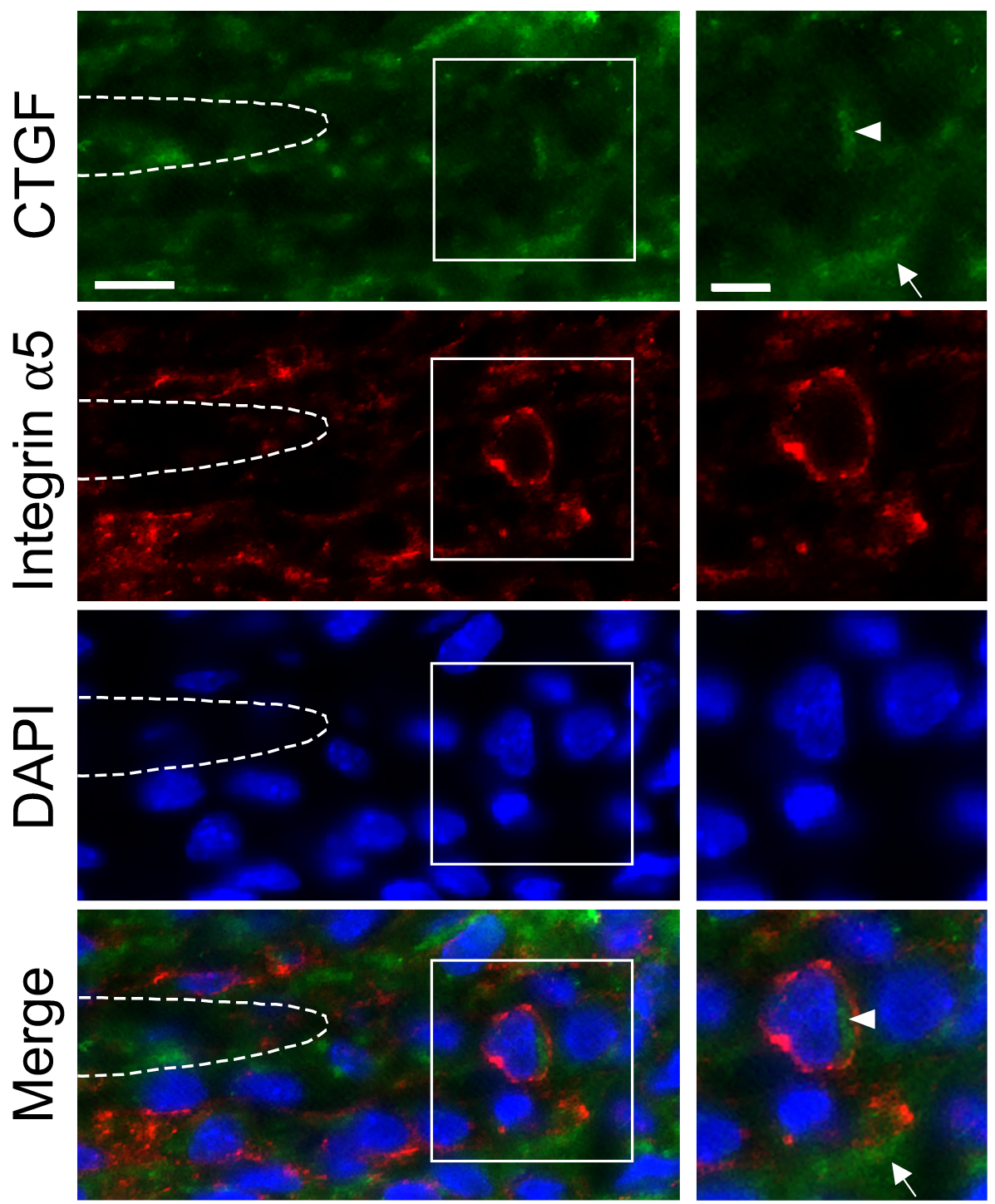


**Supplementary Figure S2. Expression of CTGF and integrin α5 at the osteogenic front during tensile force-induced bone formation.** Expression of CTGF (green) and integrin α5 (red) at the osteogenic front at 7 days after application of tensile force in our mouse model was analyzed by immunohistochemistry. The dotted line in the image of immunohistochemistry indicates the border of mineralized bone. Right panels are magnified images of the square area in the left panels. Arrows indicate CTGF expression in extracellular matrix at the osteogenic front. Arrowheads indicate CTGF expression inside integrin α5^+^ cell. Scale bar = 10 µm in left panels, and 5 µm in right panels.

**
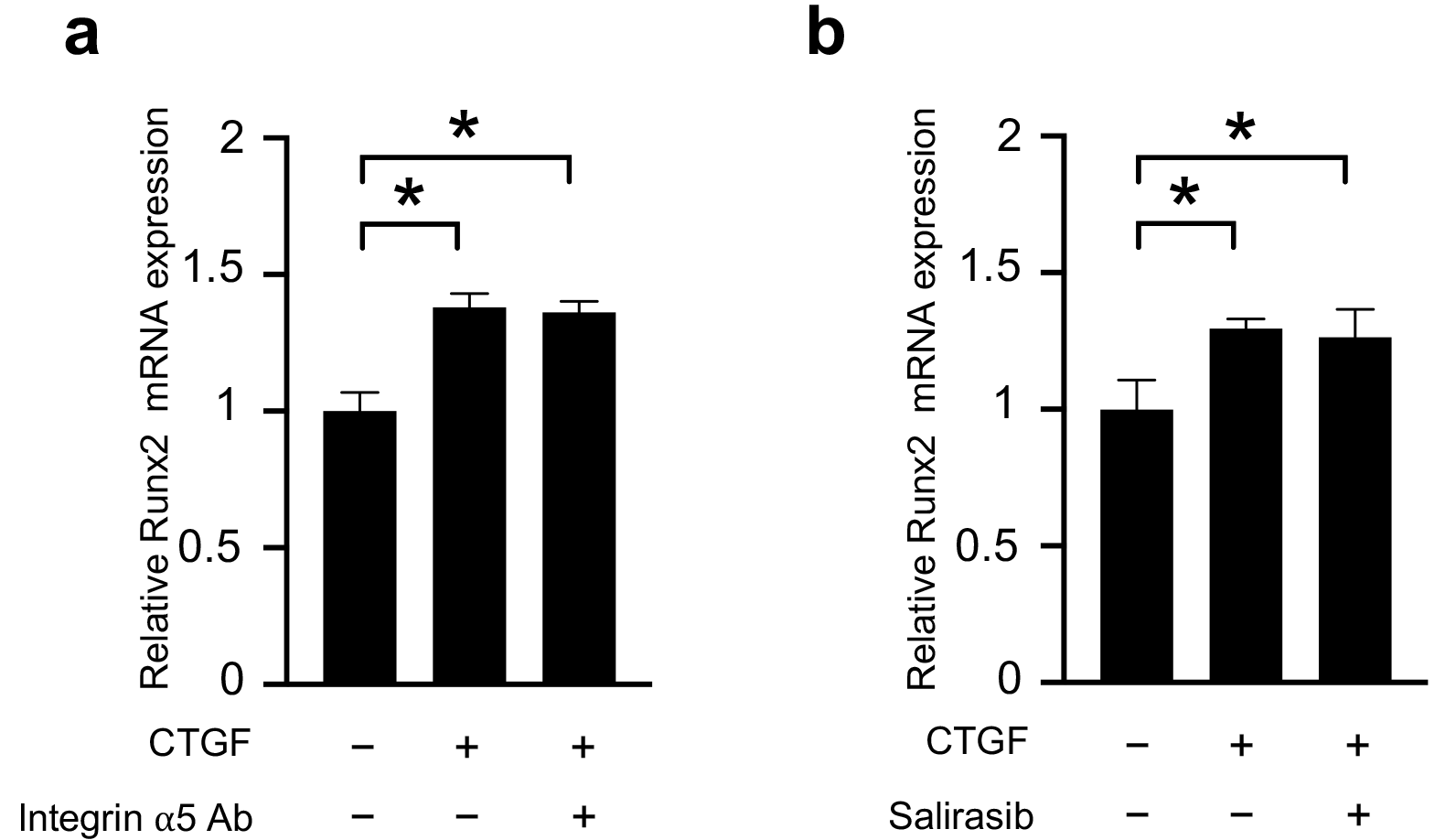
**

**Supplementary Figure S3. Neutralizing integrin α5 antibody and salirasib did not significantly affect CTGF-induced Runx2 mRNA expression in MC3T3-E1 cells.** MC3T3-E1 cells were seeded onto 24-well plates at 2 × 10^4^ cells/well. The cells were treated with 50 ng/ml CTGF and either 5 μg/ml neutralizing integrin α5 antibody (a) or 10 μM salirasib (b) for 3 days after they reached confluence. The mRNA expression of Runx2 was analyzed by real-time PCR (n=3). Integrin α5 Ab = neutralizing integrin α5 antibody. *p < 0.05, ANOVA with Tukey-Kramer post hoc test.

**Supplementary Figure S4. Original photos corresponding to Figure 6a.** The dotted red lines show the cropped region.

| **Gene Name Primer Sequence Accession No.** |
| --- |
| Runx2 5′-GACGTGCCCAGGCGTATTTC-3′ (S) NM_001146038  5′-AAGGTGGCTGGGTAGTGCATTC-3′ (AS) |
| GAPDH 5′-TGTGTCCGTCGTGGATCTGA-3′ (S) NM_001289726  5′-TTGCTGTTGAAGTCGCAGGAG ′ (AS) |

**Supplementary Table S1. Primers used for real-time PCR.**
